# Icephobic Coating Based on Novel SLIPS Made of Infused PTFE Fibers for Aerospace Application

**DOI:** 10.3390/polym16050571

**Published:** 2024-02-20

**Authors:** Adrián Vicente, Pedro J. Rivero, Nadine Rehfeld, Andreas Stake, Paloma García, Francisco Carreño, Julio Mora, Rafael Rodríguez

**Affiliations:** 1 Engineering Department, Campus de Arrosadía S/N, Public University of Navarre, 31006 Pamplona, Spain; pedrojose.rivero@unavarra.es (P.J.R.); rafael.rodriguez@unavarra.es (R.R.); 2 Institute for Advanced Materials and Mathematics (INAMAT^2^), Campus de Arrosadía S/N, Public University of Navarre, 31006 Pamplona, Spain; 3 Departmet Paint Technology, Fraunhofer Institute for Manufacturing Technology and Advanced Materials (IFAM), 28359 Bremen, Germany; nadine.rehfeld@ifam.fraunhofer.de (N.R.); andreas.stake@ifam.fraunhofer.de (A.S.); 4 INTA-Instituto Nacional de Técnica Aeroespacial, Área de Materiales Metálicos, Ctra. Ajalvir km 4, 28850 Torrejón de Ardoz, Spain; garciagp@inta.es (P.G.); fcarpue@inta.es (F.C.); jmornog@inta.es (J.M.)

**Keywords:** electrospinning, PTFE, SLIPS, super hydrophobic, low ice adhesion

## Abstract

The development of slippery surfaces has been widely investigated due to their excellent icephobic properties. A distinct kind of an ice-repellent structure known as a slippery liquid-infused porous surface (SLIPS) has recently drawn attention due to its simplicity and efficacy as a passive ice-protection method. These surfaces are well known for exhibiting very low ice adhesion values (τice < 20 kPa). In this study, pure Polytetrafluoroethylene (PTFE) fibers were fabricated using the electrospinning process to produce superhydrophobic (SHS) porous coatings on samples of the aeronautical alloy AA6061-T6. Due to the high fluorine–carbon bond strength, PTFE shows high resistance and chemical inertness to almost all corrosive reagents as well as extreme hydrophobicity and high thermal stability. However, these unique properties make PTFE difficult to process. For this reason, to develop PTFE fibers, the electrospinning technique has been used by an PTFE nanoparticles (nP PTFE) dispersion with addition of a very small amount of polyethylene oxide (PEO) followed with a sintering process (380 °C for 10 min) to melt the nP PTFE together and form uniform fibers. Once the porous matrix of PTFE fibers is attached, lubricating oil is added into the micro/nanoscale structure in the SHS in place of air to create a SLIPS. The experimental results show a high-water contact angle (WCA) ≈ 150° and low roll-off angle (αroll-off) ≈ 22° for SHS porous coating and a decrease in the WCA ≈ 100° and a very low αroll-off ≈ 15° for SLIPS coating. On one hand, ice adhesion centrifuge tests were conducted for two types of icing conditions (glaze and rime) accreted in an ice wind tunnel (IWT), as well as static ice at different ice adhesion centrifuge test facilities in order to compare the results for SHS, SLIPs and reference materials. This is considered a preliminary step in standardization efforts where similar performance are obtained. On the other hand, the ice adhesion results show 65 kPa in the case of SHS and 4.2 kPa of SLIPS for static ice and <10 kPa for rime and glace ice. These results imply a significant improvement in this type of coatings due to the combined effect of fibers PTFE and silicon oil lubricant.

## 1. Introduction

Ice formation and accretion on surfaces represents a challenge, especially on aircraft [1], wind turbines [2], among others [3]. For example, in aviation, at first glance, one could assume that ice development is limited to specific regions and the winter months. Nevertheless, ice formation can in fact occur anywhere, even during warm seasons. However, the probability of encountering ice will be higher in the vicinity of large bodies of water, on mountain ranges, or in areas close to fronts (warm, cold, and occluded) as a consequence of increased altitude and humidity. Furthermore, in contrast to the summer, faster ice accumulations will occur in the winter at lower elevations (less than 4.5 km above ground level). Additionally, as we approach the poles, ice development will happen at lower altitudes [4].

Over the last decades, numerous studies have been carried out to solve icing problems. One of the most widely used methods has been the application of active systems. However, in order to obtain a lower consumption of these, it is necessary to design and develop passive icephobic surfaces and coatings. The term “icephobicity” is defined as the ability to prevent ice formation (anti-icing) or remove ice from a surface (de-icing) [5,6]. On one side, the anti-icing surfaces use different approaches, such as the surface’s capacity to reject incoming water droplets in cold climates, resulting in the absence of water and, consequently, ice [7,8] and the surface’s capacity to postpone or prevent ice nucleation and frost formation, thereby extending the duration of the liquid phase of water on the surface [9,10,11,12]. On the other side, the deicing surfaces have low adhesion strength (<100 kPa) to ice [13,14,15,16]. However, in cold and harsh environments, the formation of ice or frost is typically unavoidable over an extended period, so extremely low values of ice adhesion are required.

Through the combination of materials with low surface energy and a certain roughness, the Superhydrophobic surfaces (SHS) display an ultra-high WCA. The trapped air inside the textured surface reduces the water droplet’s interaction with the solid surface [17]. Ice adhesion values as low as 50 kPa have been reported for these surfaces. Nevertheless, a growing amount of research indicates that even these moderately low ice adhesion values cannot be maintained due to condensation and frost formation [6,11,18,19]. Therefore, the anti/de-icing performance of the SHS depends on the ability to remain both water droplets and ice in a Cassie–Baxter state [20].

The PTFE surfaces presents strong bonds between the fluorine and carbon atoms, leading to hydrophobicity, low roll-off angles, low hysteresis, immersion stability, low ice adhesion and mechanical properties.

The PTFE surfaces presents strong bonds between the fluorine and carbon atoms, result in minimal ice adhesion, immersion stability, low roll-off angles, hydrophobicity and mechanical properties [21,22,23,24,25]. However, PTFE is difficult to process due to its insolubility in common solvents and high crystalline melting point (337 °C) [26]. Several fabrication techniques include sandpaper roughening, microetching, laser-plasma X-ray, laser/femtosecond laser, 3D printing, laser ablation, hot embossing and spray coating among others [27,28] have been reported. Recently, the electrodynamic techniques have been added to these methods, such as electrospinning [27,28,29], which is an easy, economical, and flexible technique that uses an electrically charged jet of polymer solution for various fiber manufacturing scales [30].

On account of the deficiency of SHS, the SLIPS are composed of a porous solid substrate with a rough texture, where a lubricant is infused to provide a homogenous, smooth contact free of defects. It could be compared to SHS where the lubricant replaces the air trapped between the substrate and the water. To date, a similar approach of electrospun SLIPS was developed by TAS et al. [31], obtaining remarkable, low-ice adhesion values (<1 kPa), which is lower than most of the reported studies using lubricants (τice = 16 kPa) [12,32,33]. The lubricant-impregnated surface achieves low ice adhesion by minimizing the contact angle hysteresis and tilt angle on the surface through the formation of a low surface energy (typically highly fluorinated) lubricating free-oil layer, such as PTFE. Nevertheless, for long-term stable performance of SLIPS, even under harsh environmental conditions, it is necessary to retain the lubricant oil in the SHS porous surface [32,34].

Over the past few decades, a wide variety of test techniques have been developed to evaluate ice accretion and adhesion measurements, addressing various ice types, levels of intricacy, and ice-removal methods [35]. The use of an icing wind tunnel (IWT) is one of the best methods to reproduce real icing conditions as a complement of static ice, obtaining the three principal types of ice through the control of different variables in icing labs [36]. Three varieties of ice can arise from freezing phenomena: glaze ice, typically acquired in high liquid water content (LWC) conditions, characterized by irregularly shaped and transparent crystals. On the other hand, rime ice usually forms in low LWC conditions, creating opaque crystalline layers composed of a porous structure with air trapped and with lower density than glaze; mixed ice demonstrates properties that lie between those of glaze and rime ice [37]. In assessing ice adhesion, the centrifuge tests demonstrated robustness, ease of handling and accessibility. These test devices provide reproducible results within a reasonable timeframe [38,39,40,41]. They are generally used in a comparative manner, using reference or benchmark surfaces (e.g., aluminum, steel, unmodified coatings) to identify improvements (reduced ice adhesion) for the new developed materials.

In this work, a SLIPS was prepared through infusion of a SHS made from pure PTFE. This PTFE porous coating was obtained by means of electrospinning a dispersion of PTFE nanoparticles inside a very small amount of ultra-high molecular weight of polyethylene oxide (PEO). Later on, the PEO was removed, and microfibers were formed via thermal treatment. In order to maximize the icephobic properties, the SHS porous membrane was infused with lubricating oil to obtain SLIPS. As a result, an accurate identification of the electrospun samples in terms of thickness, surface morphology and chemical composition has been obtained. Finally, the SLIPS and SHS coatings were compared to analyze wettability properties and ice-adhesion performance. The surfaces were characterized in terms of ice adhesion measurements carried out with a centrifuge test using different centrifuge test facilities. The glaze and rime ice were accreted in IWT at INTA and the static ice at INTA and IFAM to compare both centrifuge test methodologies. Therefore, the purpose of this work is the development of icephobic surfaces, focusing on a novel approach using Slippery Liquid-Infused Porous Surfaces (SLIPS) created from infused Polytetrafluoroethylene (PTFE) fibers. In addition, this research implies a preliminary step in standardization efforts, where similar performance is obtained in different ice lab facilities.

## 2. Experimental Procedure

### 2.1. Materials and Reagents

The flat substrates of AA6061-T6 aluminum alloy (AL-USTOCK, Vitoria, Spain) for membrane preparation (dimensions 76 × 56 × 2 mm^3^) and centrifuge test at IFAM and INTA (dimension 30 × 30 × 3 mm^3^) were polished and cleaned with isopropanol achieving a roughness lower than Ra = 0.8 µm prior to material application.

Poly(ethylene oxide) (PEO, (-CH_2_CH_2_O-)n, Mw ≈ 5,000,000 g/mol), N,N-Dimethylformamid (DMF, ReagentPlus^®^, purity ≥ 99%) and lubricant silicon oil ([-Si(CH_3_)_2_O-]_n_, viscosity 1000 cSt (25 °C)) were acquired from Sigma-Aldrich (Saint Luis, MO, USA). CHEMOURS Fluoropolymers (Gijon, Spain) supplied the aqueous PTFE dispersion (Teflon^®^ PTFE DISP 30, PTFE-1). The dispersion contained 60 wt% nanoparticles, 34 wt% distilled water, and 6 wt% polyoxyethylene alkylether, a non-ionic surfactant.

### 2.2. Fabrication Techniques

#### 2.2.1. Solution and Dispersion Preparation

The PEO was dissolved in a mixture of DMF and distilled water with a weight ratio 1:1 ($w_{DMF}:w_{H_{2}O}$), resulting in a PEO solution of 2 wt%. Thus, a PEO solution was obtained at room temperature and under strong stirring (600 rpm) for 24 h. Then, the PTFE-1 dispersion was added in a weight ratio 98:2 ($w_{PTFE-1}:w_{PEO}$) of PTFE solid content respect to PEO solid content, resulting in a homogeneous PTFE-PEO solution at room temperature and under weak stirring (200 rpm) for 12 h.

The PTFE dispersion (PTFE-1) was mixed in an ethanol and distilled water mixture with a volume ratio 1:2 ($V_{Eth}:V_{H_{2}O}$). As a result, after 10 min at room temperature and slow stirring (50 rpm), a PTFE dispersion containing 30 wt% PTFE nanoparticles (PTFE-2) was produced. For the preparation of the solutions and dispersions, a scale and a pipette was used to determine the quantity of each of the reagents to be added.

#### 2.2.2. Electrospinning (ES) and Electrospraying (SP)

An Electrospinning Machine (Doxa Microfluidics^®^ S.D., Malaga, Spain) was used. It allows control over several operating parameters, including flow rates, evaporation distance, electrical currents, droplet diameter deposition, and Taylor Cone visualization. It is used for Electrospinning & Electrospraying, where a double polarization voltage is applied (needle/collector) to remove the “fly out” nanoparticles and enhance both procedures. The negative electrode was a flat collector, while the positive electrode was a needle with an outer/inner diameter of 0.9/0.6 mm. Both processes were completed at room temperature 24 ± 3 °C and 38 ± 5% relative humidity (RH).

##### Coating Fabrication Process

**Figure 1:**
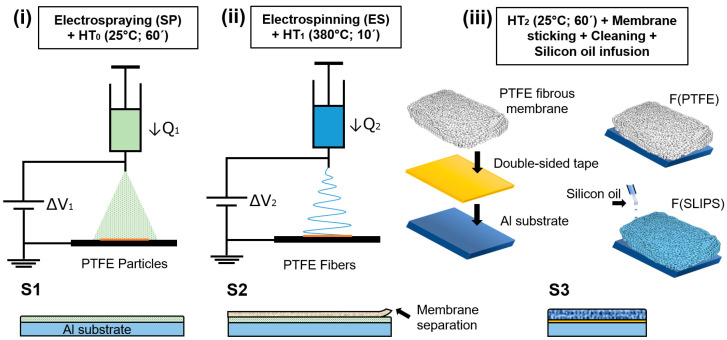
Schematic illustration of the fabrication methods to produce F(PTFE) and F(SLIPS) composite samples through S1, S2 and S3 steps: (**i**) electrospraying with PTFE-2 dispersion and HT_0_; (**ii**) S1+ electrospinning a PEO-PTFE fibrous coating and HT_1_; (**iii**) S2 + HT_2_ + membrane sticking + cleaning + silicon oil infusion.

The F(PTFE) and F(SLIPS) coatings were prepared onto the aluminum substrates using a three-step method that included electrospraying, electrospinning, and silicon oil infusion. The steps of the manufacturing process are presented in Table 1 and Figure 1.

(i) Electrospraying + HT_0_

In the first step S1 (Figure 1i) the PTFE-2 dispersion was loaded into 10 mL syringe located vertically on the electrohydrodynamic system and used as surface pre-treatment, in order to obtain a homogeneous layer of PTFE particles as an anti-adherent barrier to separate the next layers from the substrate. The parameters used during the electrospraying process are summarized on Table 2 (SP) and the coating thickness was 31 ± 5 µm. After the electrospraying process the surface was rested at room temperature for 1 h.

(ii) Electrospinning + HT_1_

After resting, the PTFE-PEO solution was loaded into the syringe for the preparation of the electrospun membrane with the parameter configuration shown on Table 2 (ES). Then, the sample was placed in a high-temperature muffle at 380 °C (HT_1_) for 10 min to remove PEO and melt the PTFE particles together, achieving pure PTFE fibrous in S2.

(iii) HT_2_ + Membrane sticking + Silicon oil infusion

Once in step S3, the coating is cooled to room temperature, it was separated from the substrate and a PTFE fibrous membrane was obtained. Then, a small piece of the membrane was mounted in the test substrate using a double-sided tape and removing the excess of material along the edges through a sharp cutter, obtaining the F(PTFE) sample. As soon as the membrane was fully stuck to the substrate, the sample was cleaned with isopropanol and after 15 min of drying, the surface was ready to lubricant infusion. Finally, the electrospun sample was infused with lubricant silicon oil along the entire surface, and afterwards, the sample was kept in a 45° tilted plate overnight to get rid of excess oil (Figure 1ii), obtaining F(SLIPS) sample in step S3.

### 2.3. Characterization Techniques

The thickness of the samples was assessed through the magnetic induction method at multiple locations, and the average value was determined following DIN EN ISO 2808:2019 standards, using the byko-test 8500 P Fe/NFe instrument (Byk Gardner, Geretsried, Germany) [42]. On one side, the resultant surface morphology of the samples was analyzed by a field- emission scanning electron microscopy (FE-SEM, LEO 1530, Zeiss GmbH, Jena, Germany). On the other side, the FE-SEM images were measured using the ImageJ program in order to determine the sample F(PTFE)’s average fiber diameter (Df) and particle size (Ps). An Attenuated Total Reflection Fourier-Transform Infrared (ATR-FTIR) spectroscopy study was conducted on the samples to determine their present functional groups. An Alpha II spectrophotometer (Bruker, Ettlingen, Germany) was utilized to measure the samples’ spectra in the 400–4000 cm^−1^ range.

Wettability tests were conducted using the Drop Shape Analyzer DSA 100S (Krüss GmbH, Hamburg, Germany), following the pertinent specifications (DIN EN ISO 19403-2) [43]. The water contact angle (WCA) was obtained using a water droplet delivered at a rate of 0.2 μL/s with a total volume of 6.0 μL. The sliding angle or roll-off angle (WSA) was determined using a tilt speed of 60°/min and a water droplet volume of 20 μL, where the advancing and receding angles of the water droplet moved at least 1 mm from the starting point [43]. By calculating the difference between the advancing and receding angles, contact angle hysteresis (CAH) for this sliding angle was obtained. The tilting method was chosen for its consistency in delivering reliable results for fresh samples.

Ice adhesion centrifuge tests were conducted in the Fraunhofer IFAM ice lab (Bremen, Germany) and in the National Institute of Aerospace technology of Spain (INTA). Both facilities have the centrifuge adhesion test (CAT) and icing wind tunnel (IWT) integrated inside a cold climate chamber, offering temperature stability during the ice accretion, the transport to the ice adhesion rig, and the ice adhesion test. The ambient temperature was kept constant to the accretion temperature for each condition (−8 °C or −5 °C). And also during the pre-conditioning and adhesion tests. This temperature was chosen because it is low enough to avoid the possibility of unstable icing conditions (these may occur close to 0 °C). Samples for testing were pre-conditioned to this test temperature before the ice formation procedure started. With respect to the ice types and velocities, four distinct scenarios were established for the assessment of the ice adhesion (see Table 3). The same area (9 cm^2^) was covered in all cases accreting the same mass of ice (close to 3 g).

**Figure 2:**
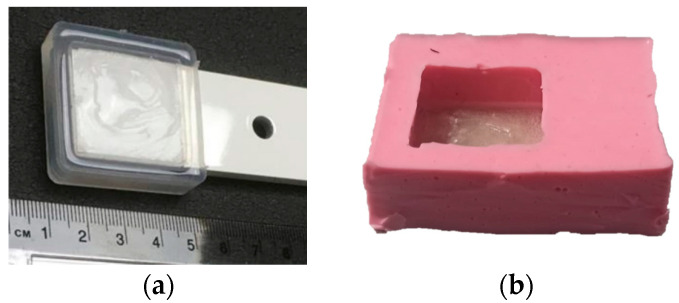
Test sample preparation with a silicon mold for static ice at IFAM (**a**) and INTA (**b**).

Static ice in Table 3, was produced at INTA and IFAM, where each test sample was placed into a silicone mold that facilitated precise ice formation in the needed region (Figure 2a,b). The mold was filled with three milliliters of de-ionized water, which was then let to freeze onto the test surface. The silicone mold was taken out after 90 min, and before the sample was put into the centrifuge, it was kept at the test temperature for at least 15 min without being mechanically disturbed.

**Figure 3:**
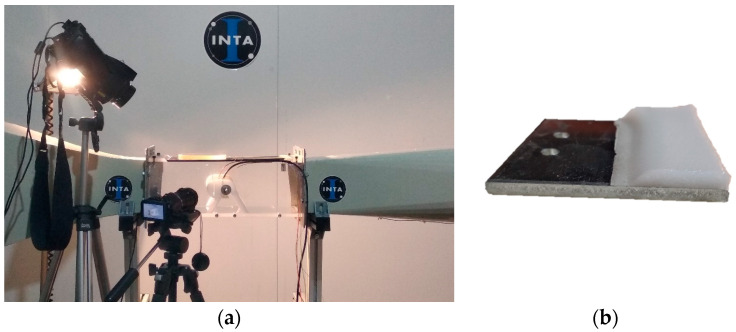
Test section of the icing wind tunnel (IWT) at INTA (**a**); specimen holder for ice wind tunnel insertion for impact ice formation (**b**).

For impact ice, the pre-conditioned test samples were evaluated at INTA, placing the test surface inside the IWT test chamber perpendicular to the airflow for a period of time specified (Figure 3a). The sample was extracted from the ice wind tunnel after impact ice formation and weighed at a constant temperature of −5 °C. Then the test sample was located and fixed in the CAT for testing of the ice adhesion. All the specimens were handled with care to avoid heat transmission from the operator to the surface. One benefit of impact icing in IWTs over in-mold icing is the ability to replicate in-flight icing conditions, where the water droplets in the ice wind tunnel segment contact the surface and quickly freeze at the test surface.

Different types and forms of ice (Table 4) were formed as a result of the four ice accretion scenarios (Table 3). In the instance of static ice, a precisely defined region formed compact glaze ice. Condition 1’s impact ice also had a clear glazed appearance, whereas Condition 2 created rime ice, both conditions forming a rough surface.

Ice adhesion tests were conducted using two distinct custom-made centrifuges, each equipped with a modified rotor designed to place and fasten the prepared test samples at INTA and IFAM. Both systems were described in detail in previous studies [44,45].

According to the equation presented in [46], the ice adhesion or shear stress needed to detach the ice from the surface was correlated to the rotational speed of the centrifuge rotor when the ice hit the centrifuge wall.

The reference material used in this study was “PTFE-tape” (extruded Polytetrafluoroethylene PTFE film tape 5490; 3M Deutschland GmbH, Neuss, Germany), which was applied on primed test samples according to the supplier’s specifications. The film thickness of the tape was 90 µm.

Two replicates of each material were consecutively tested until the full coating degradation. In addition several “PTFE-tape” for comparison purposes were tested, where the mean value and standard deviation were calculated.

## 3. Results and Discussion

### 3.1. Samples Thickness and Surface Morphology

To analyze the surface morphology and thus determine its structure, the coating thickness, fiber diameter and particle size of the F(PTFE) samples are evaluated and summarized in Table 5.

**Figure 4:**
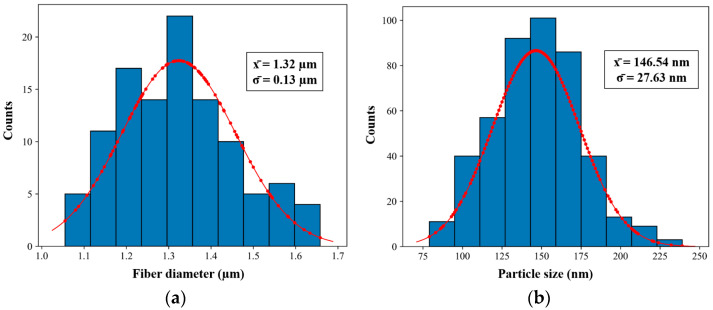
Histograms of the diameter distribution (**a**) and particle size (**b**) of the fiber sample F(PTFE).

The fiber diameters and particles size in the Figure 4 histogram are presented with diameter size (a) and particle size (b) for samples F(PTFE) having a log normal distribution shape, typically observed in nanoparticles’ size distribution that leads to the average values presented on Table 5.

**Figure 5:**
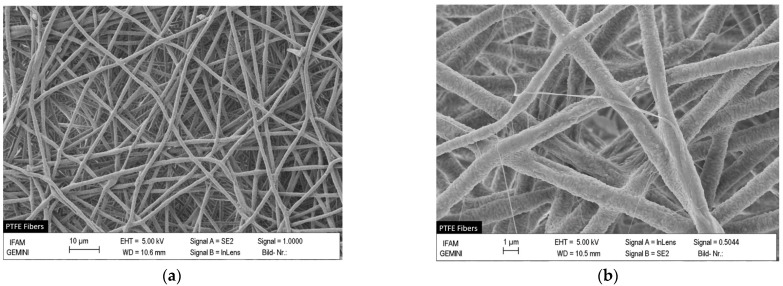

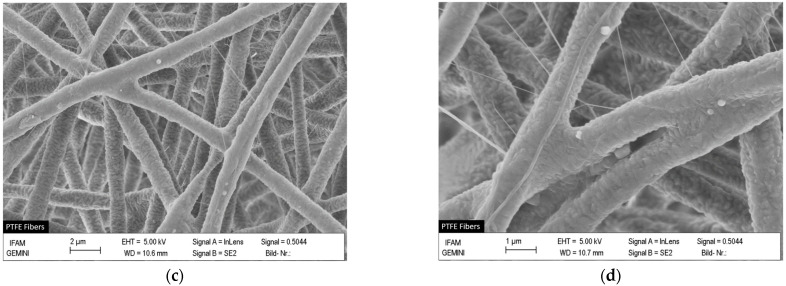
Scanning electron microscopy (SEM) images of the sample surface morphology F(PTFE) at the scale of 10 µm (**a**), 2 µm (**b**,**c**) and 1 µm (**d**).

The SEM images of Figure 5a,b show a network of uniform PTFE fibers composed of PTFE nanoparticles melted together after ES + HT_1_ procedure, as can be seen in detail in Figure 5d. The extremely low amount of PEO in the PEO/PTFE composite fibers and PEO decomposition induced by HT_1_, results in minimal content of carbonized residues, around the PTFE particles (see Figure 5c,d).

### 3.2. Chemical Composition

**Figure 6:**
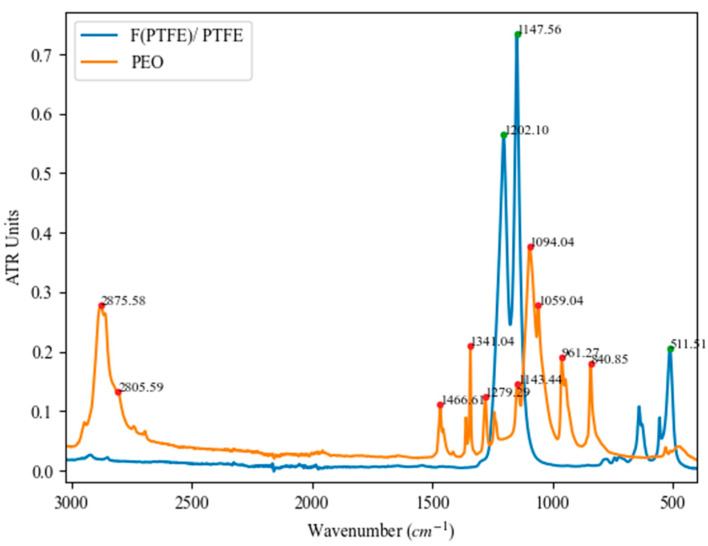
FTIR spectra of the samples F(PTFE) and PEO.

In order to identify the chemical composition of the samples surface, an FTIR study was performed. In Figure 6, FTIR spectra of the electrospun sample F(PTFE) show strong absorption peaks at 1202.52 cm^−1^ and 1147.63 cm^−1^, which correspond to the C-F bond and C-C bond of the PTFE, respectively. The peak at 640 cm^−1^ could be attributed to the absorption of CF_2_ wagging [47].

A very low quantity of PEO was added to the electrospinning solution to aid fiber formation. In Figure 6, the typical triplet peak of pure PEO is observed from 1143 to 1058 cm^−1^. The peak at 1094.97 cm^−1^ has been attributed to typical stretching vibration of ether group C-O-C in PEO spectra [48]. In addition, pure PEO exhibits a peak at 2875.61 cm^−1^ of asymmetric CH_2_ stretching. Finally, as can be observed, F(PTFE) does not present any contributions of PEO because of the HT_1_ degradation process on the low PEO content present in the fibers. Moreover, the F(PTFE) shows the same spectra as the PTFE reference sample [49].

### 3.3. Wetting Properties

With the intention to determine the samples’ wettability properties, the static and dynamic water angles are studied. On one hand, the F(PTFE) sample has shown a superhydrophobic behavior. The combined effect of water repellency by the previously mentioned fluoride functional groups, along with a hierarchical micro/nano structure based on microfibers composed of nanoparticles, results in a Cassie–Baxter state [50,51]. The presence of air entrapped inside the gaps, makes the penetration of the liquid into the texture of the surface, difficult due to capillary forces [52,53], leading to a significant improvement in the static and dynamic wettability properties (see Table 6). On the other hand, the F(PTFE) surface is ideal for the development of SLIPS, as this surface is easy to wet and exhibits excellent chemical stability with the lubricating liquid. Moreover, the porous structure of F(PTFE) traps the lubricating liquid which is immiscible with water.

Thus, to reduce the adhesion forces, a lubricating film was applied. The silicon oil was used as lubricant for SLIPS, filling the grooves of the electrospun porous surface and increasing the capillary forces, obtaining the F(SLIPS) sample. As can be appreciated in Table 6, the F(SLIPS) has lower WCA than the F(PTFE) due to reduction in roughness [54]. In addition, the roll-off angle ($\alpha_{roll-off}$) in the F(PTFE), the water sliding angle (WSA) in the F(SLIPS) and the contact angle of hysteresis (CAH) for both samples were measured to check the dynamic wetting properties. The sample F(SLIPS), despite has lower contact angle, obtained a higher water droplet mobility compared with lubricant-free structure. This implies a lower CAH and WSA as a result of the low water interaction of F(SLIPS) respect to F(PTFE) (see Table 6).

### 3.4. Ice Adhesion Performance

#### 3.4.1. Static Ice at IFAM and INTA

**Figure 7:**
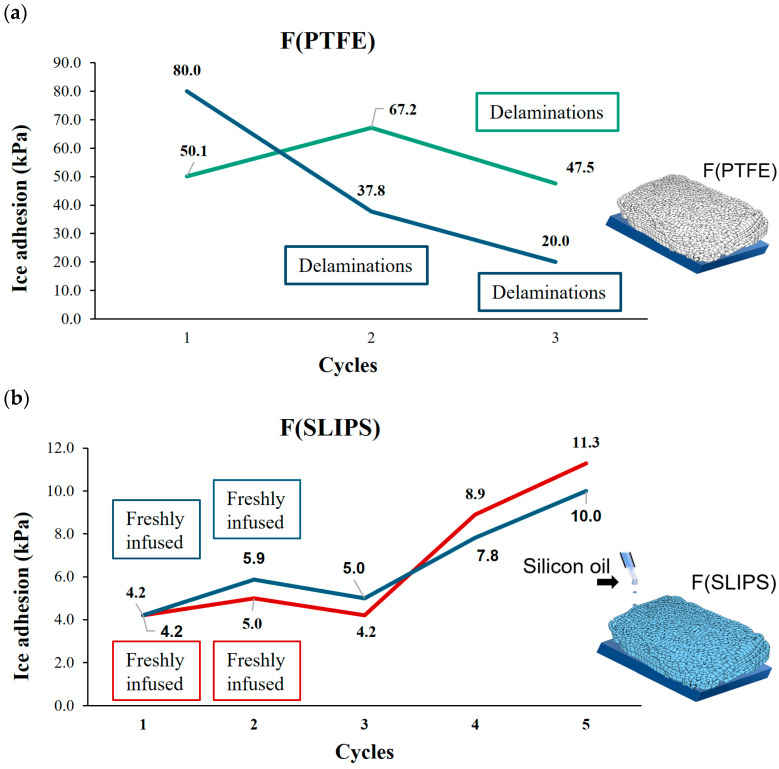
Ice adhesion test results for static ice and repeated cycles for the samples F(PTFE) (**a**) and F(SLIPS) (**b**) obtained at IFAM.

Once the wettability properties have been analyzed, the icing behavior is studied. In this subsection, the static ice adhesion properties at IFAM and INTA are evaluated. On one hand, static ice at IFAM tests were obtained by using two replicates per cycle, expressed in Figure 7. For F(PTFE), ice adhesion was low (80 kPa, 50 kPa, respectively) and further decreased in the subsequent cycles. After the second cycle, material delamination became obvious, indicating low cohesion properties between the fiber layers. For F(SLIPS), ice adhesion was even lower (4.2 kPa, 5.0 kPa) for the freshly infused layers. This performance remained after the second cycle, but without renewing the infusion liquid. In the following cycles 4 and 5, where reinfusion was not applied, the ice adhesion strength increased, but was still at a very low level.

**Figure 8:**
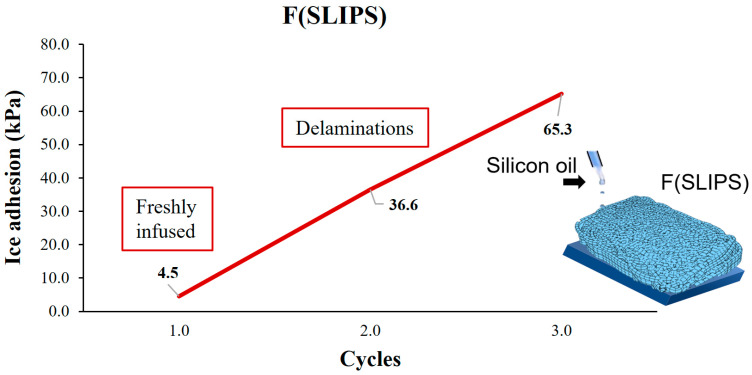
Ice adhesion test results for static ice starting from pristine conditions and repeated cycles for the samples F(SLIPS) obtained at INTA.

On the other hand, static ice tests at INTA were obtained in Figure 8. In this case, just the F(SLIPS) ice adhesion was analyzed, showing a low ice adhesion value (4.5 kPa) for the freshly infused layers. However, after the second cycle, clear signs of material delamination was evident. The loss of lubricant liquid in the porous structure during the first cycle led to the formation of Wenzel ice, resulting in cohesive failure of the fibers under shear stress and a decrease in the lubricating layer. In the following third cycle without renewing the infusion liquid, the ice adhesion strength increased twofold, but ice adhesion remained low.

**Figure 9:**
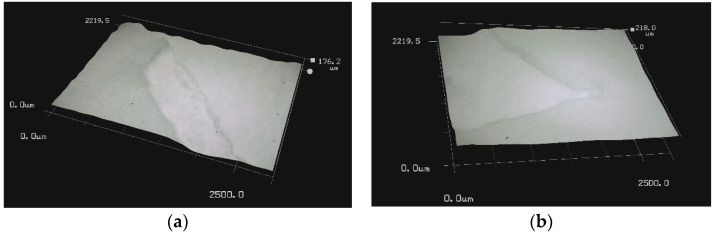
Optical microscopy images of the surface delamination for F(PTFE) (**a**) and F(SLIPS) (**b**).

When observing the surface of the coating after the test, signs of delamination of the coating due to cohesive breaks are found due to Wenzel ice (see Figure 9a,b).

**Figure 10:**
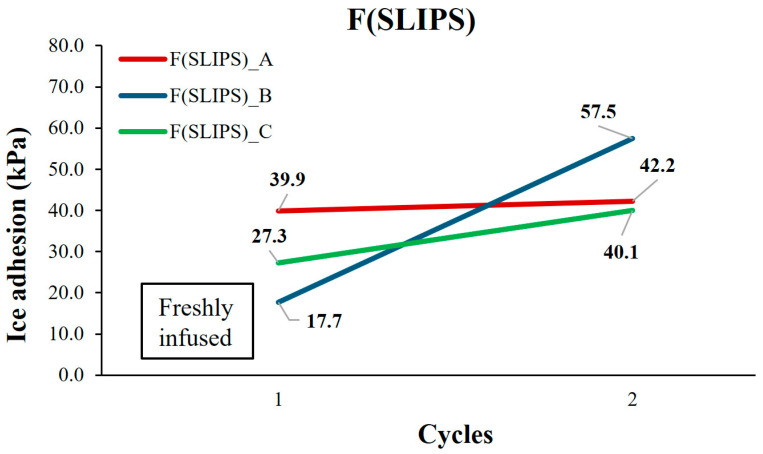
Ice adhesion test results for static ice for the delaminated samples F(SLIPS) obtained at INTA.

After the delamination the specimens were reinfused several times using the fibers still attached to the adhesive layer and re-tested to check if the very low adhesion performance was recovered (see Figure 10).

The ice adhesion value in the initial tests after reinfusion decreased compared to that obtained with the aged specimen but are higher than those obtained with the pristine F(SLIP) coating. In the next second cycle, where the infusion liquid was not applied, the ice adhesion strength increased, observing a slight average rise despite having a thin layer of fibers attached to the adhesive layer.

#### 3.4.2. Impact Ice in IWT at INTA (Glaze and Rime)

**Figure 11:**
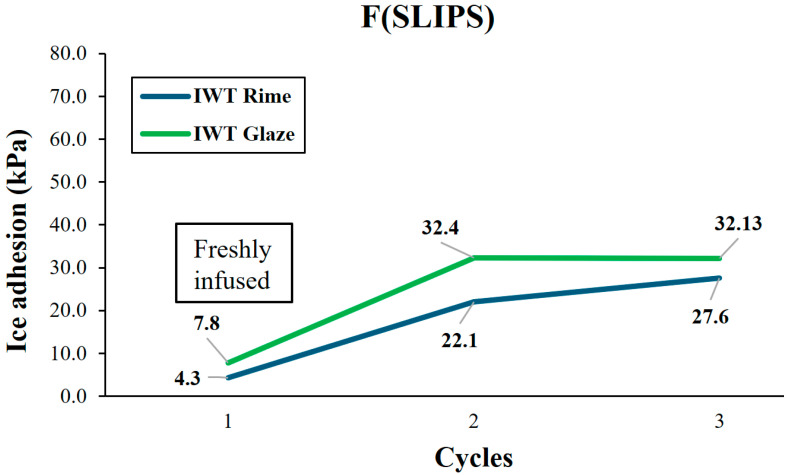
Ice adhesion test results for IWT rime ice and IWT glaze ice starting from pristine conditions in sample F(SLIPS) obtained at INTA.

Next, in this subsection the ice adhesion for both rime and glaze ice in IWT ice accretion for F(SLIPS) at INTA are studied. The results obtained were similar, showing ultra-low ice adhesion (<10 kPa) in the first test and increasing to around 30 kPa in the followings (see Figure 11). This behavior could be explained considering that the SLIPS retains a layer of oil that would remain on the surface of the coating, leading to a two-phase water/oil contact in the first freezing and, after the removal of the oil surface layer in that first test, becoming a new three-phase interface water/PTFE fibers/oil contact showing a higher adhesion to ice.

#### 3.4.3. Comparison with Reference Materials

In order to be able to compare the values obtained with reference materials, two common materials are tested. At INTA, testing was conducted on the AA6061 T6 in its as-received condition, alongside PTFE tape 3M 5490 for both static and IWT ice. At IFAM, only PTFE tape 3M 5490 was tested with static ice. The results obtained from these tests are presented in Table 7.

Finally, to check if the low adhesion of F(SLIPS) is only due to the effect of the oil, a layer of oil is placed on an AA6061 substrate and ice is formed inside a mold (static) to prevent the oil from dripping off. The adhesion result obtained was 183 ± 52 kPa, lower than that of bare aluminum but far away from the adhesion value of SLIPS. This highlights the need to have a porous matrix to retain the oil.

The durability of the specimens once they lose lubrication is very limited, with a life of about 2 to 3 tests before the cohesive breakage of the PTFE fibers. This effect can be avoided by reinfusing the lubricant before delamination of the layers occurs.

Once the upper delaminated layers are detached, it is observed that there are still fibers adhered to the adhesive layer, so reinfusion is attempted. It can be observed in the results that the adherence of the ice to the observed surface—ice adhesion—is greater than when the infusion occurs on the virgin specimen, probably because it has a greater capacity to retain the lubricant.

## 4. Conclusions

In the present study, a SLIPS was successfully prepared through a pure PTFE fibrous coating. This fibrous surface was fabricated by electrospinning technique by using PEO/PTFE solution, adding a very low amount of PEO to PTFE nanoparticle dispersion, followed with heat treatment (380 °C). The surface morphology of the sample was analyzed by SEM, showing uniform PTFE micro fibers composed of nano particles with homogeneous density. In addition, this hierarchical micro/nano structure in combination with fluorine groups (CF_2_) presents in PTFE fibers after the complete decomposition of low PEO content, resulting in a superhydrophobic behavior (WCA > 150°) with excellent water mobility and leading to a low roll-off angle (<25°), respectively.

A SLIPS was obtained, filling the micro/nanoscale structure with lubricating oil instead of air in the SHS. On one side, the WCA value has shown a hydrophobic behavior generated by a decrease in roughness, and on the other side, the water mobility has increased with very low contact angle hysteresis and water sliding angle (CAH = 16°, WSA < 15°).

In addition, the ice adhesion centrifuge test was carried out for two different types of icing conditions (glaze and rime) simulated in an ice wind tunnel at INTA, as well as static ice at different ice adhesion centrifuge test facilities (INTA and IFAM) in order to compare the results for F(SLIPS). The static ice adhesion results at IFAM shows 65 kPa in the case of F(PTFE) and 4.2 kPa of F(SLIPS) for the freshly infused layers. Moreover, the ice adhesion for static ice in freshly F(SLIPS) at INTA and IFAM show similar ultra-low ice adhesion results. However, an increase in the ice adhesion has been obtained for the next cycles without renewing the infusion liquid, especially at INTA, where some delamination occurs in contracts to F(SLIPS) at IFAM, where it has still remained at a very low level (11.3 kPa). The durability of the specimens once lubrication is lost is very limited, due to the cohesive breakage of the PTFE fibers caused by Wenzel ice. This effect can be avoided by reinfusing the lubricant before delamination of the layers occurs or after the delamination using the rest of fibers still attached to the adhesive layer, having enough capacity to retain the lubricant. The results obtained in F(SLIPS) for both rime and glaze ice in IWT at INTA were similar, showing ultra-low ice adhesion (<10 kPa) in the first test and increasing to around 30 kPa in the following tests due to a removal of lubricant.

Finally, the static ice adhesion values in both facilities (INTA and IFAM) for a reference material (PTFE tape) and the F(SLIPS) show a small difference, achieving a first step for standardization.

According to this study, it can be concluded that icephobic performance is directly related to the ability of the fibrous layer to retain lubricant. As lubricant is lost, damage accumulates in the fibrous layers due to Wenzel ice, and thus its ability to hold lubricant is reduced. The fibrous layer in contact with the adhesive layer experiences a higher resistance to delamination by Wenzel ice; however, its ability to retain lubricant will be lower. Finally, in the upper layers of fibers, where the adhesion due to the cohesive forces between the fibrous layers is lower, the damage caused by Wenzel ice is higher. Therefore, to improve this coating, it is key to increase the cohesive forces between fibers, through increased compaction between fibrous layers and reduced pore size which helps to increase capillary forces and thus retain the lubricant. So, the cohesion of fibers layer, retention and replacement of the lubricant in the porous matrix is one of the aspects to improve and continue researching in this type of coatings.

## Figures and Tables

**Table 1:** Summary of the methods, composition and heat treatments of each step (Sx).

Step (Sx)	Composition	Procedures	Heat Treatment (HT)		
				Temp (°C)	Time (min)
S1	PTFE-2	SP + HT_0_	HT_0_	25	60
S2	PEO + PTFE	S1 + ES + HT_1_	HT_1_	380	10
S3	Si-oil	S2 + HT_2_ + St + Inf	HT_2_	25	60

**Table 2:** Configuration of the electrospinning and electrospraying parameters.

Parameters	SP	ES
Applied voltage (needle/collector) (KV)	8.90/−3.00	2.72/−3.05
Flow rate (mL/h)	0.5	1.0
Deposition time/sample (min)	30	60
Distance	15	20

**Table 3:** Summary table of the icing test conditions.

	Static Ice IFAM	Static Ice INTA	Impact Ice (IWT, INTA)	
			Condition 1	Condition 2
Wind speed (m/s)	――	――	70	70
Temperature (°C)	–8 ± 0.5°	–8 ± 0.5°	–5 ± 0.5°	–15 ± 0.5°
Liquid water content (g/m^3^)	――	――	0.5	0.5
Median volumen diameter (µm)	――	――	40	20
Icing duration	90 min	90 min	4 min	4 min
Ice área (cm^2^)	9	9	9	9
Resulting ice mass (g)	3	3	3	3

**Table 4:** Ice types for ice adhesion tests.

Static Ice	Static Ice	Impact Ice (IWT, INTA)	
IFAM	INTA	Condition 1	Condition 2
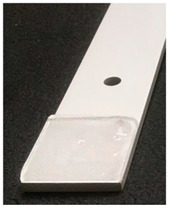	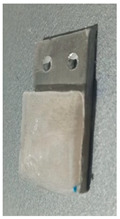	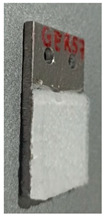	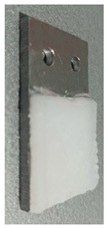
glaze ice, compact	glaze ice, compact	glaze ice	rime ice

**Table 5:** Summary of coating thickness, average fiber diameter and particle size of F(PTFE).

Sample	Thickness (µm)	Df (µm)	Ps (nm)
F(PTFE)	224 ± 7	1.32 ± 0.13	146.54 ± 27.63

**Table 6:** The water contact angle (WCA), the roll-off water angles ($\alpha_{\mathrm{roll}-\mathrm{off}}$), the water sliding angle (WSA) and the contact angle of hysteresis (CAH) of the F(PTFE) and F(SLIPS) samples.

	F(PTFE)	F(SLIPS)
WCA	150.3±2.3°	98.4±0.9°
$\alpha_{\mathrm{roll}-\mathrm{off}}$/WSA	22.8±3.6°	14.6±1.1°
CAH	62.0±1.0°	16.0±2.9°

**Table 7:** Ice adhesion values of the reference materials (AA6061 and PTFE tape) for IWT ice at INTA and Static ice at INTA and IFAM.

	IWT Ice (kPa)	Static Ice (kPa)
AA6061-INTA	212±14	231±24
PTFE tape-INTA	102±19	91±26
PTFE tape-IFAM	―	131±18

## Data Availability

Data are contained within the article.
